# Supplementary material for: Association of Daily Physical Activity and Sedentary Behaviour with Protein Intake Patterns in Older Adults: A Multi-Study Analysis across Five Countries
Source: Nutrients. 2021 Jul 27;13(8):2574. doi: 10.3390/nu13082574 (PMC8398631; doi:10.3390/nu13082574)
Supplement: Supplementary file 1 [file nutrients-13-02574-s001.zip › nutrients-1281268-supplementary.pdf]

Supplementary material

Methods

Protein intake patterns-eating occasions

In the *Newcastle 85+ Study* and *LiLACS NZ*, to describe the timing of protein intake, protein intake (absolute and as a proportion of total protein intake) was calculated for seven eating occasions dividing the 24-hour period into three-hour slots (from 05:30 to 23:29) plus six hours 23:30-5:29, as previously [1]. The eating occasions were subsequently classified as breakfast, morning snack, lunch, afternoon snack, dinner, evening snack, and overnight snack).

In *DNFCS* and *INRAN-SCAI* the pre-defined meal occasions were: breakfast, morning snack, lunch, afternoon snack, dinner, evening snack. In *FINDIET*, participants reported the meal occasion themselves (breakfast, lunch, supper, dinner, snack, drink, other) and these were then reclassified into the same six groups as in *DNFCS* and *INRAN-SCAI*, including information on the time of consumption for the classification of 'snacks', 'drinks' and 'other' into morning snack, afternoon snack and evening snack. Supper was reclassified as follows: when participants reported lunch, dinner and supper or dinner and supper, supper was reclassified as evening snack. When participants reported breakfast and supper or supper only, supper was reclassified as dinner.

Socioeconomic, Lifestyle, and Health Factors

Education:
Newcastle 85+ Study: Education was self-reported and defined as years spent in full-time education: up to 9 years, 10-11 years, and 12 or more.

The *Life and Living to Advanced Age Cohort Study New Zealand (LiLACS NZ)*: Education was self-reported and defined as low: primary school or no schooling; intermediate: secondary school; high: occupational trade or tertiary.

National dietary surveys: Education was defined according to self-reported highest attained education or years of education and categorised into three survey-specific categories. The *Dutch National Food Consumption Survey* (*DNFCS)*: low: no education, primary or lower vocational education or advanced elementary education; intermediate: intermediate vocational or higher secondary education; high: higher vocational education or university. The *Finnish FINDIET surveys* (*FINDIET):* years of education were categorised into low, intermediate and high according to tertiles by sex and birth year. The *third Italian National Food Consumption Survey* (*INRAN-SCAI)*: low: no title, primary or lower secondary education; intermediate: upper secondary education; high: university degree.

Poverty/household income:

In the *Newcastle 85+ study*, the Index of Multiple Deprivation (IMD) was used to define level of poverty in current area of residency and was categorised into tertiles (poor areas, intermediate group areas, affluent areas) [2]. The IMD is a composite, weighted measure of multiple deprivations in seven domains (income, employment, health and disability, education, housing and services, living environment, and crime) for small geographic areas of about 1,500 inhabitants [3]. In *LiLACS NZ* a similar measure was used, the NZ deprivation index [4] classified into tertiles (low, mid, high).

Household income was available in *DNFCS* and *FINDIET* only and classified into low, high and unknown. In *DNFCS* household income was considered to be low when net income was <1700 euros per month. In *FINDIET* household income was low when yearly income before tax deduction was < 30.000 euros.

Living arrangements:

In *the Newcastle 85+ Study* and *LiLACS NZ*, living arrangement was categorised as living alone or not living alone.

In the *national surveys* self-reported information on marital status was used to classify living arrangement into living together (married, cohabiting) and not living together (single, separated or divorced, widow).

Lifestyle factors

Newcastle 85+ Study and *LiLACS NZ*: Lifestyle factors were self-reported and included current smoking (yes/no) and current alcohol intake (yes/no).

National dietary surveys: Smoking was self-reported and classified into never, former or current smokers (*DNFCS* and *FINDIET*) or non-smokers, current smoker (*INRAN-SCAI*). For *INRAN-SCAI* there were a relatively large number of missing values for smoking (and education), therefore, for *INRAN-SCAI* only, these missing values were included as a separate category in the analyses.

BMI was calculated as kg/m^2. Body weight and height were measured in the *Newcastle 85+ Study, LiLACS NZ*, *DNFCS* and *FINDIET*, but self-reported in *INRAN-SCAI*.

Results

Participant characteristics

Table S1. Differences in selected characteristics according to physical activity level of community-dwelling older adults in the UK (Newcastle 85+), New Zealand (LiLACS NZ), Netherlands (DNFCS), Finland (FINDIET) and Italy (INRAN-SCAI)

	Physical activity level				
	**All**	**Low**	**Intermediate**	**High**	**p-value**
Newcastle 85+	N=721	N=184	N=335	N=202	
Age (years)	85.5 ± 0.4	85.5 ± 0.4	85.5 ± 0.4	85.5 ± 0.5	0.49
Sex (% male, n)	40.1 (289)	37.5 (69)	32.8 (110)	54.5 (110)	<0.0001
Body Mass Index (kg/m^2)	24.5 ± 4.4	24.4 ± 4.4	24.7 ± 4.5	24.2 ± .93	0.53
Living alone (%, n)	57.7 (416)	58.3 (95)	63.0 (208)	55.9 (113)	0.24
Education (≥12 years) (%, n)	12.5 (90)	10.4 (19)	13.3 (27)	13.4 (27)	0.77
Index of multiple deprivation (%, n)					0.07
- Poor areas	25.1 (181)	20.1 (37)	25.4 (85)	29.2 (59)	
- Intermediate	50.6 (365)	48.9 (90)	53.1 (178)	48.0 (97)	
- Affluent areas	24.3 (175)	30.9 (57)	21.5 (72)	22.8 (46)	
Smokers (%, n)	5.9 (43)	8.7 (16)	5.1 (17)	5.0 (10)	0.19
Alcohol (%, n)	63.5 (458)	54.4 (100)	62.4 (209)	73.8 (149)	<0.0001
Adjusted body weight (kg)	63.9 ± 8.2	62.7 ± 7.5	63.3 ± 8.1	65.7 ± 8.8	0.001
Chronic disease count (≥3) (%, n)	39.8 (287)	52.2 (96)	40.3 (135)	27.7 (56)	0.0001
Disability (1-6) (%, n)	55.6 (401)	38.7 (70)	70.6 (235)	47.5 (96)	0.0001
Swallowing problems (%, n)	57.8 (416)	63.9 (117)	59.4 (199)	49.5 (100)	0.012
LiLACS NZ Non-Māori	N=353	N=123	N=120	N=110	
Age (years)	85.5 ± 0.5	85.7 ± 0.5	85.4 ± 0.5	85.6 ± 0.5	0.005
Sex (% male, n)	47.9 (169)	43.9 (54)	42.5 (51)	58.2 (64)	0.033
Body Mass Index (kg/m^2)	26.7 ± 4.1	26.7 ± 5.1	26.7 ± 3.6	26.7 ± 3.4	0.75
Living alone (%, n)	50.9 (163)	52.5 (53)	52.7 (59)	47.7 (51)	0.71
Education[a] (%, n)					0.08
- low	17.4 (61)	17.2 (21)	22.9 (27)	11.8 (13)	
- intermediate	55.7 (195)	54.9 (67)	57.6 (68)	54.5 (60)	
- high	26.7 (94)	27.9 (34)	19.5 (23)	33.6 (37)	
Index of deprivation (%, n)					0.25
- High deprivation	38.8 (137)	41.5 (51)	40.0 (48)	34.5 (38)	
- Mid	37.4 (132)	30.9 (38)	41.7 (50)	40.0 (44)	
- Low deprivation	23.8 (84)	27.6 (34)	18.3 (22)	25.5 (28)	
Smokers (%, n)	1.7 (6)	3.3 (4)	0.8 (1)	0.9 (1)	0.25
Alcohol (%, n)	70.5 (249)	58.5 (72)	78.3 (94)	75.5 (83)	0.01
Adjusted body weight (kg)	67.1 ± 8.7	65.9 ± 9.2	66.8 ± 8.2	68.7 ± 8.4	0.02
Chronic disease count (≥3) (%, n)	64.3 (227)	70.7 (87)	69.2 (83)	51.8 (57)	0.02
Disability (1-6) (%, n)	69.4 (245)	50.0 (61)	77.6 (90)	90.4 (94)	<0.01
Swallowing problems (%, n)	19.1 (61)	25.7 (26)	16.1 (18)	15.9 (17)	0.12

LiLACS NZ Māori	N=183	N=58	N=57	N=68	
Age (years)	83.3 ± 2.6	83.7 ± 3.0	83.3 ± 2.6	83.1 ± 2.4	0.72
Sex (% male, n)	42.1 (77)	36.2 (21)	35.1 (20)	52.9 (36)	0.07
Body Mass Index (kg/m^2)	28.7 ± 5.6	30.7 ± 6.7	27.7 ± 5.7	27.9 ± 4.2	0.05
Living alone (%, n)	35.5 (65)	45.7 (21)	45.8 (22)	37.9 (22)	0.64
Education[a] (%, n)					0.24
- low	27.2 (49)	36.8 (21)	25.0 (14)	20.9 (14)	
- intermediate	60.6(109)	56.1 (32)	62.5 (35)	62.7 (42)	
- high	12.2 (22)	7.0 (4)	12.5 (7)	16.4 (11)	
Index of deprivation (%, n)					0.09
- High deprivation	60.7 (111)	56.9 (33)	64.9 (37)	60.3 (41)	
- Mid	25.1 (46)	31.0 (18)	28.1 (16)	17.6 (12)	
- Low deprivation	14.2 (26)	12.1 (7)	7.0 (4)	22.1 (15)	
Smokers (%, n)	7.1 (13)	3.4 (2)	12.3 (7)	5.9 (4)	0.16
Alcohol (%, n)	54.4 (99)	38.6 (22)	66.7 (38)	57.4 (39)	0.01
Adjusted body weight (kg)	65.9 ± 8.0	65.4 ± 6.4	64.0 ± 7.7	67.9 ± 8.8	0.05
Chronic disease count (≥3) (%, n)	56.3 (103)	69.0 (40)	52.6 (30)	48.5 (33)	0.02
Disability (1-6) (%, n)	68.9 (124)	39.7 (23)	76.4 (42)	88.1 (59)	<0.01
Swallowing problems (%, n)	16.7 (25)	20 (9)	18.8 (9)	12.3 (7)	0.522
DNFCS	N=738	N=32	N=126	N=580	
Age (years)	77.1 ± 5.2	81.5 ± 6.4	77.4 ± 5.3*	76.8 ± 5.1*	<0.0001
Sex (% male, n)	50.5 (373)	50.0 (16)	57.9 (73)	49.0 (284)	0.19
Body Mass Index (kg/m^2)	27.4 ± 3.8	29.8 ± 6.4	27.8 ± 3.8	27.2 ± 3.6*	0.0033
Married/living together (%)	61.5 (454)	53.1 (17)	61.9 (126)	61.9 (359)	0.61
Education[a] (%)					0.95
- Low	55.5 (408)	62.5 (20)	54.8 (69)	55.3 (319)	
- Intermediate	21.9 (161)	18.8 (6)	22.2 (28)	22.0 (127)	
- High	22.6 (166)	18.8 (6)	23.0 (29)	22.7 (131)	
Household income (%, n)					0.33
- Low	35.8 (264)	50.0 (16)	31.0 (39)	36.0 (209)	
- High	36.9 (272)	25.0 (8)	41.3 (52)	36.6 (212)	
- Unknown	27.4 (202)	25.0 (8)	27.8 (35)	27.4 (159)	
Smoking (%, n)					0.0065
- Never	34.3 (253)	28.1 (9)	20.6 (26)	37.6 (218)	
- Former	55.4 (409)	62.5 (20)	68.3 (86)	52.2 (303)	
- Current	10.3 (76)	9.4 (3)	11.1 (14)	10.2 (59)	
FINDIET	N=867	N=143	N=544	N=180	
Age (years)	68.8 ± 2.8	69.4 ± 2.8	68.8 ± 2.8	68.4 ± 2.7*	0.0033
Sex (% male, n)	50.5 (438)	46.2 (66)	50.2 (273)	55.0 (99)	0.28
Body Mass Index (kg/m^2)	28.2 ± 4.7	30.4 ± 5.8	28.1 ± 4.1*	26.8 ± 3.7*^	<0.0001
Married/living together (%)	71.8 (622)	67.8 (97)	71.1 (386)	77.2 (139)	0.14
Education[a] (%)					0.012
- Low	30.8 (261)	38.0 (52)	31.5 (167)	23.5 (42)	
- Intermediate	35.3 (299)	32.9 (45)	36.7 (195)	33.0 (59)	
- High	33.9 (287)	29.2 (40)	31.8 (169)	43.6 (78)	
Household income (%, n)					0.0011
- Low	53.6 (465)	62.2 (89)	54.2 (295)	45.0 (81)	
- High	40.8 (354)	30.1 (43)	39.9 (217)	52.2 (94)	
- Unknown	5.5 (48)	7.7 (11)	5.9 (32)	2.8 (5)	

Smoking (%, n)					0.011
- No	58.2 (496)	51.8 (73)	58.7 (314)	61.6 (109)	
- Former	31.8 (271)	32.6 (46)	30.7 (164)	34.5 (61)	
- Current	10.1 (86)	15.6 (22)	10.7 (57)	4.0 (7)	
INRAN-SCAI	N=510	N=67	N=214	N=229	
Age (years)	74.5 ± 7.3	76.5 ± 8.2	75.0 ± 7.8	73.6 ± 6.4*	0.0076
Sex (% male, n)	39.6 (202)	50.8 (34)	44.9 (96)	31.4 (72)	0.0021
Body Mass Index (kg/m^2)	25.7 ± 4.1	27.0 ± 5.8	25.4 ± 3.5*	25.7 ± 3.9	0.0195
Married/living together (%)	50.8 (259)	49.3 (33)	53.3 (114)	48.9 (112)	0.85
Education[a] (%)					
- Low	71.0 (362)	67.2 (45)	69.6 (149)	73.4 (168)	0.057
- Intermediate	15.1 (77)	10.5 (7)	14.0 (30)	17.5 (40)	
- High	8.8 (45)	13.4 (9)	9.4 (20)	7.0 (16)	
- Unknown	5.1 (26)	9.0 (6)	7.0 (15)	2.2 (5)	
Household income (%, n)	N/A	N/A	N/A	N/A	-
Smoking (%, n)					
- No	85.9 (438)	77.6 (52)	84.6 (181)	89.5 (205)	0.0229
- Current	10.8 (55)	13.4 (9)	11.7 (25)	9.2 (21)	
- Unknown	3.3 (17)	9.0 (6)	3.7 (8)	1.3 (3)	

*Presented as mean ± standard deviation for continuous variables and % (n) for categorical variables. * Differs from those with a low physical activity level p<0.05. ^ Differs from those with an intermediate physical activity level. [a]: LiLACS NZ: low: no schooling or primary; intermediate: secondary; high: occupational trade or tertiary. DNFCS: low: no education, primary or lower vocational education or advanced elementary education; intermediate: intermediate vocational or higher secondary education; high: higher vocational education or university.*
FINDIET: years of education were categorised into low, intermediate and high according to tertiles by age and birth year.
INRAN-SCAI: low: no title, primary or lower secondary education; intermediate: upper secondary education; high: university degree.

Table S2. Differences in selected characteristics according to sedentary behaviour of community-dwelling older adults in Finland (FINDIET) and Italy (INRAN-SCAI)

	Sedentary behaviour			p-value
	**<4 hrs/day**	**4-6 hrs/day**	**>6 hrs/day**	
FINDIET (n=798)	N=261	N=360	N=177	
Age (years)	68.9 ± 2.8	68.9 ± 2.8	68.4 ± 2.8	0.15
Body Mass Index (kg/m^2)	27.9 ± 4.3	28.0 ± 4.3	28.5 ± 4.7	0.28
Sex (% male, n)	42.5 (111)	53.1 (191)	58.2 (103)	0.0028
Married/living together (%, n)	69.7 (182)	75.8 (272)	71.2 (126)	0.22
Education (%, n)				
Low	32.8 (84)	29.3 (103)	25.4 (45)	0.0007
Intermediate	35.9 (92)	39.0 (137)	26.0 (46)	
High	31.3 (80)	31.6 (111)	48.6 (86)	
Household income (%, n)				
Low	62.3 (163)	51.9 (187)	44.6 (79)	0.0005
High	32.6 (85)	44.7 (161)	53.1 (94)	
Unknown	5.0 (13)	3.3 (12)	2.3 (4)	
Smoking (%, n)				
No	67.8 (173)	55.3 (197)	48.9 (86)	0.0012
Former	23.5 (60)	33.7 (120)	40.3 (71)	
Current	8.6 (22)	11.0 (39)	10.8 (19)	
INRAN-SCAI (n=493)	N=81	N=152	N=260	
Age (years)	78.3 ± 7.7	75.1 ± 7.3*	72.8 ± 6.5*^	<0.0001
Body Mass Index (kg/m^2)	25.9 ± 4.9	25.8 ± 3.7	25.6 ± 4.1	0.82
Sex (% male, n)	35.8 (29)	38.8 (59)	41.5 (108)	0.63
Married/living together (%, n)	30.9 (25)	54.0 (82)	55.8 (144)	0.0003
Education (%, n)				
Low	75.3 (61)	70.2 (106)	77.4 (195)	0.51
Intermediate	17.3 (14)	19.2 (29)	13.5 (34)	
High	7.4 (6)	10.6 (16)	9.1 (23)	
Household income (%, n)	N/A	N/A	N/A	-
Smoking (%, n)				
No	92.6 (75)	92.1 (139)	85.7 (222)	0.072
Current	7.4 (6)	8.0 (12)	14.3 (37)	

*Presented as mean ± standard deviation for continuous variables and % (n) for categorical variables. * Differs from those with <4 hrs sedentary behaviour per day p<0.05. ^ Differs from those with 4-6 hrs sedentary behaviour per day.*

Table S3. Energy and protein intake according to physical activity tertile in community-dwelling older adults from the Newcastle 85+ Study, LiLACS NZ, and national surveys (adjusted for age and sex)

		Physical activity level			
	**All**	**Low**	**Intermediate**	**High**	**p-value**
Newcastle 85+ Study	N=721	N= 184	N= 335	N= 202	
Energy intake (kcal/day)	1613.0 ± 18.8	1617.6 ± 37.5	1625.6 ± 24.9	1838.0 ± 38.5	0.0001
Protein intake					
- grams/day	64.3 ± 0.8	59.7 ± 1.5	62.6 ± 1.1	71.2 ± 1.8	0.0001
- g/kg/ aBW/day	1.00 ± 0.01	0.95 ± 0.02	0.99 ± 0.02	1.09 ± 0.03	0.0013
- g/day main meals only	42.4 ± 0.8	37.4 ± 1.5	42.1 ± 1.1	47.4 ± 1.8	0.0003
- g/day breakfast	7.5 ± 0.3	6.9 ± 0.02	7.1 ± 0.3	8.6 ± 0.5	0.15
Low protein (<0.8 g/kg aBW/day) (%, n)	27.6 (199)	32.1 (59)	27.8 (93)	23.3 (47)	0.15
LiLACS NZ Non-Māori	N=353	N=123	N=120	N=110	
Energy intake (kcal/day)	1765.1 ± 29.5	1679.7 ± 52.5	1761.7 ± 49.1	1864.3 ± 50.3	0.012
Protein intake					
- grams/day	71.3 ± 1.3	68.8 ± 2.4	69.5 ± 1.9	76.0 ± 2.2	0.010
- g/kg/ aBW/day	1.1 ± 0.02	1.0 ± 0.03	1.0 ± 0.03	1.1 ± 0.03	0.047
- g/day main meals only	57.0 ± 1.2	53.4 ± 2.1	56.1 ± 1.9	62.0 ± 1.9	0.003
- g/day breakfast	10.8 ± 0.3	10.2 ± 0.56	10.7 ± 0.61	11.5 ± 0.63	0.29
Low protein (<0.8 g/kg aBW/day) (%, n)	19.7 (67)	23.0 (26)	18.8 (22)	17.3 (19)	0.54
LiLACS NZ Māori	N=183	N=58	N=57	N=68	
Energy intake (kcal/day)	1667.9 ± 44.4	1566.1 ± 49.3	1544 ± 43.0	1858.6 ± 66.3	0.027
Protein intake					
- grams/day	74.8 ± 2.5	68.0 ± 3.8	70.3 ± 3.9	84.4 ± 4.9	0.047
- g/kg/ aBW/day	1.1 ± 0.04	1.1 ± 0.07	1.1 ± 0.07	1.2 ± 0.08	0.34
- g/day main meals only	56.3 ± 2.2	48.4 ± 2.8	51.6 ± 3.2	67.0 ± 4.6	0.005
- g/day breakfast	10.8 ± 0.9	9.3 ± 0.9	9.2 ± 0.9	13.3 ± 2.0	0.07
Low protein (<0.8 g/kg aBW/day) (%, n)	24.7 (39)	28.9 (13)	26.9 (14)	19.7 (12)	0.050
*DNFCS**	N=1476	N=64	N=252	N=1160	
Energy intake (kcal/day)	1974 ± 17.4	1763 ± 75	1937 ± 38	1991 ± 17	0.0083
Protein intake					
- grams/day	76.3 ± 0.7	68.8 ± 3.3	75.2 ± 1.6	76.9 ± 0.8	0.045
- g/kg/ aBW/day†	1.05 ± 0.01	0.93 ± 0.05	1.04 ± 0.02	1.06 ± 0.01	0.028
- g/day main meals only	65.1 ± 0.7	58.3 ± 3.1	64.1 ± 1.5	65.7 ± 0.7	0.055
- g/day breakfast	11.5 ± 0.2	11.0 ± 1.1	11.9 ± 0.6	11.4 ± 0.3	0.63
Low protein (<0.8 g/kg aBW/day) (%, n)†	20.0 (290)	35.7 (19)	19.7 (49)	19.3 (222)	0.033
*FINDIET**	N=1734	N=286	N=1088	N=360	

Table S3. Energy and protein intake according to physical activity tertile in community-dwelling older adults from the Newcastle 85+ Study, LiLACS NZ, and national surveys (adjusted for age and sex)

		Physical activity level			
	**All**	**Low**	**Intermediate**	**High**	**p-value**
Energy intake (kcal/day)	1653 ± 18.9	1631 ± 43	1642 ± 22	1691 ± 38	0.48
Protein intake					
- grams/day	70.2 ± 0.8	68.4 ± 1.9	69.5 ± 1.0	73.4 ± 1.7	0.091
- g/kg/ aBW/day	1.01 ± 0.01	0.98 ± 0.03	1.01 ± 0.01	1.06 ± 0.02	0.058
- g/day main meals only	55.1 ± 0.7	53.5 ± 1.7	54.7 ± 0.9	57.5 ± 1.6	0.18
- g/day breakfast	12.6 ± 0.3	11.8 ± 0.7	12.6 ± 0.4	13.4 ± 0.6	0.23
Low protein (<0.8 g/kg aBW/day) (%, n)	30.6 (530)	35.9 (103)	31.4 (341)	24.1 (87)	0.018
*INRAN-SCAI**	N=1530	N=201	N=642	N=687	
Energy intake (kcal/day)	2022 ± 25	1995 ± 63	2019 ± 35	2141 ± 35	0.023
Protein intake					
- grams/day	78.2 ± 0.9	79.5 ± 2.4	79.0 ± 1.3	80.8 ± 1.4	0.63
- g/kg/ aBW/day	1.17 ± 0.01	1.16 ± 0.04	1.16 ± 0.02	1.19 ± 0.02	0.50
- g/day main meals only	75.3 ± 0.9	76.8 ± 2.3	76.0 ± 1.3	77.8 ± 1.3	0.60
- g/day breakfast	7.1 ± 0.2	6.3 ± 0.6	6.5 ± 0.3	8.1 ± 0.3	0.001
Low protein (<0.8 g/kg aBW/day) (%, n)	15.4 (235)	15.0 (30)	16.4 (105)	13.1 (90)	0.43

*Presented as mean ± standard error for continuous variables and % (n) for categorical variables. Kruskal-Wallis test for non-normally distributed continuous variables. * Days are used as the unit of analyses; sample size based on number of recall days per survey for participants with non-missing physical activity data. † n=24 missing in DNFCS.*

Physical activity and protein intake

Table S4. Protein intake according to physical activity in community-dwelling older adults from the Netherlands (DNFCS), Finland (FINDIET) and Italy (INRAN-SCAI)

	Physical activity level			p-value
	Low	Intermediate	High	
DNFCS (n=1476 days)				
Protein intake (grams/day)				
Model 1	68.8 ± 3.3	75.2 ± 1.6	76.9 ± 0.8	0.045
Model 2	68.7 ± 3.3	75.1 ± 1.8	76.5 ± 1.0	0.055
Model 3	73.8 ± 2.5	75.3 ± 1.3	75.2 ± 0.7	0.84
Exclusion special days	75.4 ± 2.5	75.7 ± 1.3	75.5 ± 0.7	0.98
Exclusion special diets	72.9 ± 2.7	75.2 ± 1.5	75.6 ± 0.8	0.61
Protein intake (g/kg aBW/day)				
Model 1	0.93 ± 0.05	1.04 ± 0.02	1.06 ± 0.01	0.028
Model 2	0.94 ± 0.05	1.05 ± 0.03	1.07 ± 0.01	0.044
Model 3	1.04 ± 0.04	1.05 ± 0.02	1.05 ± 0.01	0.93
Exclusion special days	1.05 ± 0.04	1.06 ± 0.02	1.05 ± 0.01	0.96
Exclusion special diets	1.01 ± 0.05	1.05 ± 0.02	1.05 ± 0.01	0.67
FINDIET (n=1734 days)				
Protein intake (grams/day)				
Model 1	68.4 ± 1.9	69.5 ± 1.0	73.4 ± 1.7	0.091
Model 2	67.5 ± 2.4	68.2± 1.7	72.5 ± 2.3	0.085
Model 3	68.7 ± 1.5	69.2 ± 1.1	71.1 ± 1.5	0.29
Exclusion special days	68.6 ± 1.5	69.2 ± 1.1	70.9 ± 1.5	0.34
Protein intake (g/kg aBW/day)				
Model 1	0.98 ± 0.03	1.01 ± 0.01	1.06 ± 0.02	0.058
Model 2	0.97 ± 0.03	0.99 ± 0.02	1.06 ± 0.03	0.039
Model 3	0.98 ± 0.02	1.01 ± 0.02	1.04 ± 0.02	0.11
Exclusion special days	0.98 ± 0.02	1.00 ± 0.02	1.03 ± 0.02	0.12
INRAN-SCAI (n=1530 days)				
Protein intake (grams/day)				
Model 1	79.5 ± 2.4	79.0 ± 1.3	80.8 ± 1.4	0.63
Model 2	79.1 ± 3.3	77.8 ± 2.7	79.3 ± 2.8	0.73
Model 3	79.0 ± 1.6	77.5 ± 1.4	75.1 ± 1.4	0.0061
Exclusion special days	80.4 ± 1.7	78.1 ± 1.4	76.3 ± 1.4	0.017
Exclusion special diets	79.7 ± 1.7	78.2 ± 1.3	76.0 ± 1.4	0.014
Protein intake (g/kg aBW/day)				
Model 1	1.16 ± 0.04	1.16 ± 0.02	1.19 ± 0.02	0.50
Model 2	1.16 ± 0.05	1.15 ± 0.04	1.18 ± 0.04	0.70
Model 3	1.16 ± 0.03	1.15 ± 0.03	1.12 ± 0.03	0.13
Exclusion special days	1.19 ± 0.03	1.16 ± 0.03	1.14 ± 0.03	0.15
Exclusion special diets	1.18 ± 0.03	1.16 ± 0.03	1.13 ± 0.03	0.15

Presented as mean ± standard error. Repeated measures ANOVA, including an unstructured covariance matrix to account for within-person correlation, was used to compare differences according to physical activity level. Model 1: adjusted for age and sex. Model 2: adjusted for age, sex, educational level, marital status, and smoking. Model 3: Model 2 additionally adjusted for energy intake.

Table S5. Odds ratios (OR) and 95% CI for the association between physical activity tertiles and protein status (adequate vs. low protein*) in the Newcastle 85+ Study and LiLACS NZ

	Physical activity level		
	**Low**	**Intermediate**	**High**
Newcastle 85+ Study			
OR (95% CI)			
Model 1	1.25 (0.85-1.86)	1.0 (Ref)	0.86 (0.57-1.30)
Model 2	1.28 (0.85-1.94)	1.0 (Ref)	0.82 (0.54-1.24)
Model 3	1.03 (0.61-1.74)	1.0 (Ref)	1.07 (0.65-1.78)
Model 4	1.09 (0.61-1.95)	1.0 (Ref)	1.11 (0.65-1.91)
LiLACS NZ Non-Māori			
OR (95% CI)			
Model 1	1.16 (0.60-2.22)	1.0 (Ref)	0.89 (0.45-1.77)
Model 2	1.14 (0.55-2.37)	1.0 (Ref)	0.89 (0.42-1.86)
Model 3	1.05 (0.46-2.40)	1.0 (Ref)	1.21 (0.51-2.84)
Model 4	1.45 (0.57-3.72)	1.0 (Ref)	1.30 (0.51-3.29)
LiLACS NZ Māori			
OR (95% CI)			
Model 1	1.07 (0.44-2.62)	1.0 (Ref)	0.75 (0.30-1.83)
Model 2	0.89 (0.30-2.63)	1.0 (Ref)	0.79 (0.28-2.20)
Model 3	1.04 (0.28-3.83)	1.0 (Ref)	1.31 (0.39-4.34)
Model 4	0.97 (0.24-4.00)	1.0 (Ref)	1.34 (0.34-5.29)

**low protein: <0.8 g/kg aBW/d, adequate protein: ≥0.8 g/kg aBW/d.*

Model 1 included protein intake, sex and age (LiLACS NZ); model 2 was also adjusted for living alone, education, deprivation, smoking, and alcohol; model 3 was further adjusted for energy intake; model 4 was further adjusted for disease count, disability and swallowing problems

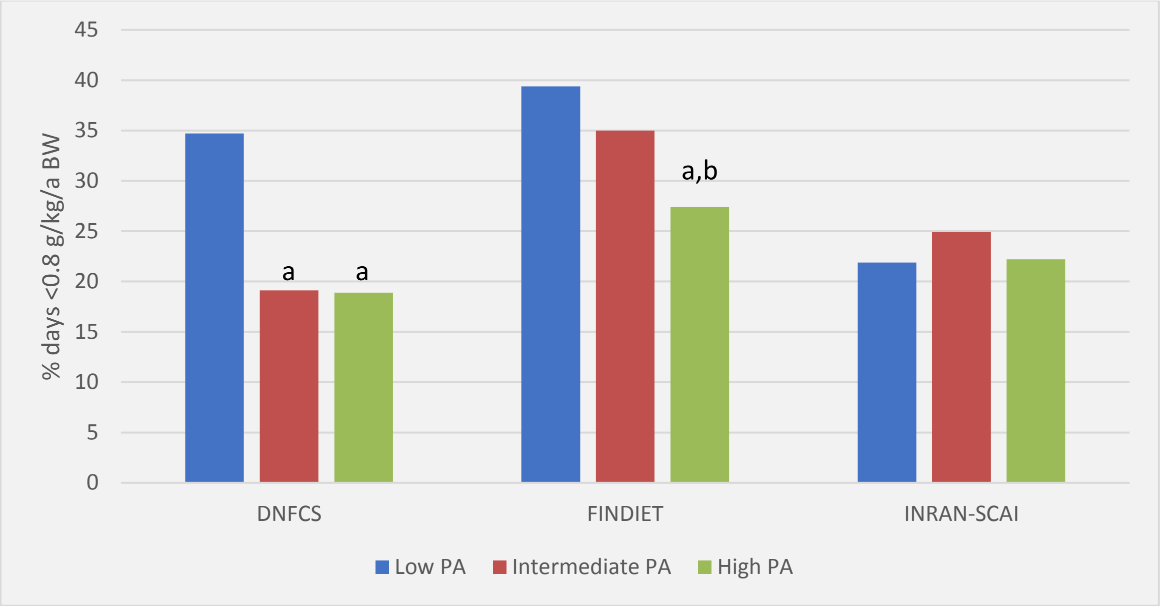

Figure S1. Percentage of days on which protein intake was below the recommended amount of 0.8 g/kg aBW according to physical activity level in older adults from the Netherlands (DNFCS n=1476), Finland (FINDIET n=1734) and Italy (INRAN-SCAI n=1530). *Adjusted for age, sex, marital status, education, (household income) and smoking.* [a] *Differs from those with a low physical activity level p<0.05.* [b] *Differs from those with an intermediate physical activity level p<0.05.*

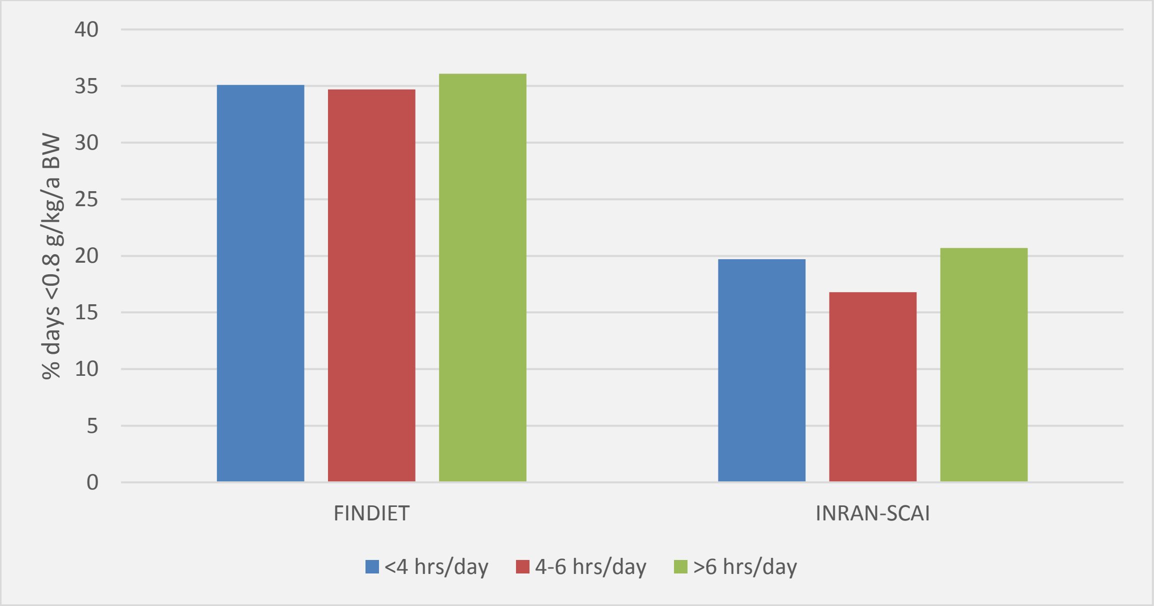

Figure S2. Percentage of days on which protein intake was below the recommended amount of 0.8 g/kg aBW according to level of sedentary behaviour in older adults from Finland (FINDIET n=1446) and Italy (INRAN-SCAI n=1479). *Adjusted for age, sex, marital status, education, (household income) and smoking.*

Table S6. Protein intake according to sedentary behaviour in community-dwelling older adults from Finland (FINDIET) and Italy (INRAN-SCAI)

		**Sedentary behaviour**			
	**All**	**<4 hrs/day**	**4-6 hrs/day**	**>6 hrs/day**	**p-value**
FINDIET (n=1596)*		N=522	N=720	N=354	
Protein intake (grams/day)	70.5 ± 0.9				
Model 1		69.7 ± 1.4	70.7 ± 1.2	70.4 ± 1.7	0.86
Model 2		67.9 ± 2.2	68.7 ± 2.1	68.5 ± 2.4	0.92
Model 3		68.6 ± 1.4	68.9 ± 1.3	68.9 ± 1.5	0.97
Exclusion special days	70.5 ± 0.9	68.6 ± 1.4	68.6 ± 1.4	68.9 ± 1.6	0.98
Exclusion special diets	N/A	N/A	N/A	N/A	
Protein intake (g/kg aBW/day)	1.02 ± 0.01				
Model 1		1.02 ± 0.02	1.02 ± 0.02	1.00 ± 0.03	0.82
Model 2		1.00 ± 0.03	1.00 ± 0.03	0.98 ± 0.03	0.88
Model 3		1.01 ± 0.02	1.00 ± 0.02	0.99 ± 0.02	0.77
Exclusion special days	1.02 ± 0.01	1.00 ± 0.02	1.00 ± 0.02	0.99 ± 0.02	0.75
Exclusion special diets	N/A	N/A	N/A	N/A	
INRAN-SCAI (n=1479)*		N=243	N=456	N=780	
Protein intake (grams/day)	78.1 ± 1.0				
Model 1		76.8 ± 2.2	81.2 ± 1.6	79.9 ± 1.2	0.28
Model 2		74.7 ± 2.6	79.2 ± 2.1	77.7 ± 1.8	0.24
Model 3		75.1 ± 1.3	76.8 ± 1.0	77.2 ± 0.9	0.24
Exclusion special days	78.8 ± 1.0	77.0 ± 1.4	77.7 ± 1.1	78.0 ± 1.0	0.77
Exclusion special diets	78.9 ± 1.0	75.9 ± 1.3	77.4 ± 1.1	78.1 ± 0.9	0.26
Protein intake (g/kg aBW/day)	1.17 ± 0.01				
Model 1		1.15 ± 0.03	1.19 ± 0.02	1.17 ± 0.02	0.59
Model 2		1.12 ± 0.04	1.16 ± 0.03	1.14 ± 0.03	0.55
Model 3		1.12 ± 0.02	1.13 ± 0.02	1.13 ± 0.02	0.93
Exclusion special days	1.18 ± 0.01	1.15 ± 0.03	1.14 ± 0.02	1.15 ± 0.02	0.92
Exclusion special diets	1.18 ± 0.01	1.14 ± 0.03	1.13 ± 0.02	1.14 ± 0.02	0.91

**Days are used as the unit of analyses. Model 1: adjusted for age and sex. Model 2: adjusted for age, sex, educational level, marital status, household income (except for INRAN-SCAI) and smoking. Model 3: Model 2 additionally adjusted for energy intake. N/A: not available.*

Physical activity and number of eating occasions providing protein

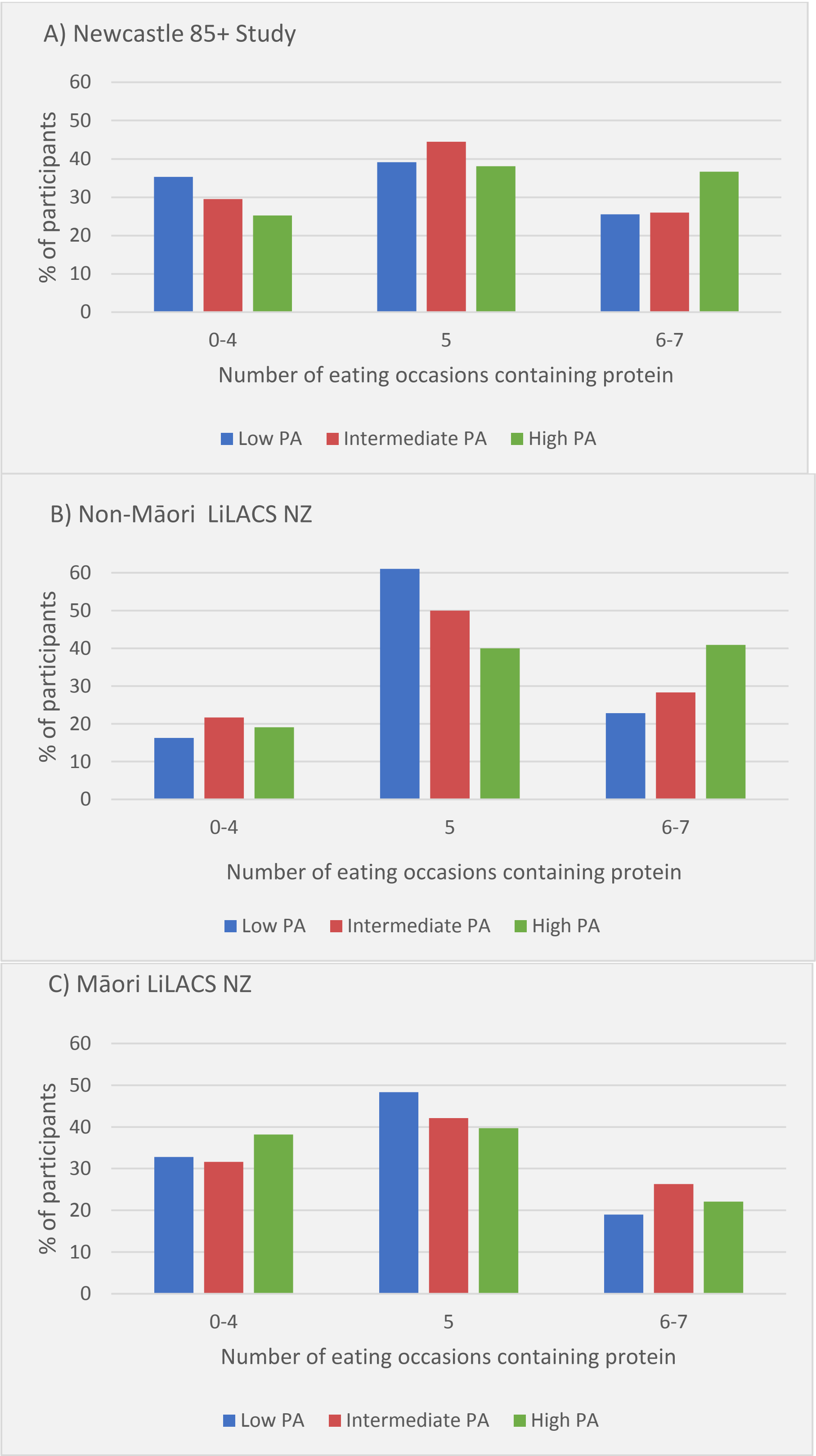

Figure S3. Percentage of participants in categories of eating occasions according to physical activity tertile in older adults of the A) Newcastle 85+ Study, B) Non-Māori, and C) Māori participants in the LiLACS NZ Study.

Table S7. Number of eating occasions providing protein according to sedentary behaviour in community-dwelling older adults from Finland (FINDIET) and Italy (INRAN-SCAI)

		Sedentary behaviour			
	**All**	**<4 hrs/day**	**4-6 hrs/day**	**>6 hrs/day**	**p-value**
FINDIET (n=1596)					
Number of eating occasions	4.5 (0.03)				
Model 1		4.6 (0.05)	4.5 (0.04)	4.5 (0.05)	0.90
Model 2		4.4 (0.07)	4.4 (0.06)	4.4 (0.07)	0.94
Model 3		4.4 (0.07)	4.4 (0.06)	4.4 (0.07)	0.92
Exclusion special days		4.4 (0.07)	4.4 (0.06)	4.4 (0.07)	0.98
Exclusion special diets		N/A	N/A	N/A	N/A
INRAN-SCAI (n=1479)					
Number of eating occasions	3.9 (0.04)				
Model 1		4.2(0.10)	4.1 (0.07)	3.7 (0.05)	<0.0001
Model 2		-	-	-	-
Model 3		-	-	-	-
Exclusion special days		3.9 (0.37)	3.8 (0.36)	3.5 (0.35)	0.0002
Exclusion special diets		-	-	-	-

*Presented as mean (standard error). *Sample size (n) refers to the number of recall days. Repeated measures ANOVA, including an unstructured covariance matrix to account for within-person correlation, was used to compare differences according to sedentary behaviour level. Model 1: adjusted for age and sex. Model 2: adjusted for age, sex, educational level, marital status, household income (except for INRAN-SCAI) and smoking. Model 3: Model 2 additionally adjusted for energy intake. N/A: not available.*

Physical activity and reaching protein thresholds

Main meals

Table S8. Odds ratios (OR) and 95% CI for the association between physical activity (PA) tertiles and frequency of main meals (0,1,2+) reaching protein thresholds in the Newcastle 85+ Study and LiLACS NZ

PA tertiles	Model 1	Model 2	Model 3	Model 4
Newcastle 85+ Study				
**20g protein**				
Low	0.79 (0.50-1.25)	0.79 (0.48-1.29)	0.81 (0.49-1.35)	0.92 (0.52-1.65)
Intermediate	1.00 (ref)	1.00 (ref)	1.00 (ref)	1.00 (ref)
High	1.14 (0.75-1.73)	1.11 (0.72-1.69)	0.96 (0.62-1.49)	0.84 (0.53-1.33)
**30g protein**				
Low	0.72 (0.43-1.20)	0.87 (0.50-1.49)	0.92 (0.53-1.61)	1.12 (0.59-2.12)
Intermediate	1.00 (ref)	1.00 (ref)	1.00 (ref)	1.00 (ref)
High	1.19 (0.76-1.85)	1.19 (0.76-1.86)	1.02 (0.64-1.64)	0.97 (0.58-1.60)
LiLACS NZ Non Māori				
**20g protein**				
Low	0.93 (0.56-1.53)	1.04 (0.60-1.83)	1.06 (0.59-1.91)	1.26 (0.67-2.37)
Intermediate	1.00 (ref)	1.00 (ref)	1.00 (ref)	1.00 (ref)
High	1.35 (0.82-2.24)	1.38 (0.81-2.34)	1.29 (0.74-2.27)	1.14 (0.63-2.08)
**30g protein**				
Low	0.81 (0.48-1.35)	0.92 (0.52-1.63)	0.95 (0.51-1.79)	0.93 (0.48-1.82)
Intermediate	1.00 (ref)	1.00 (ref)	1.00 (ref)	1.00 (ref)
High	1.28 (0.76-2.17)	1.33 (0.77-2.30)	1.14 (0.63-2.09)	1.15 (0.61-2.20)
LiLACS NZ Māori				
**20g protein**				
Low	0.72 (0.35-1.47)	0.99 (0.43-2.28)	0.79 (0.34-1.86)	0.64 (0.24-1.70)
Intermediate	1.00 (ref)	1.00 (ref)	1.00 (ref)	1.00 (ref)
High	1.89 (0.94-3.80)	1.77 (0.81-3.88)	1.23 (0.54-2.78)	1.45 (0.60-3.50)
**30g protein**				
Low	0.93 (0.43-1.98)	1.38 (0.57-3.38)	1.10 (0.41-2.91)	1.07 (0.37-3.08)
Intermediate	1.00 (ref)	1.00 (ref)	1.00 (ref)	1.00 (ref)
High	2.11 (1.02-4.36)	2.09 (0.91-4.80)	1.43 (0.57-3.58)	1.45 (0.55-3.79)

Model 1 included main meals threshold, sex and age (LiLACS only); model 2 was also adjusted for living alone, education, deprivation, smoking, and alcohol; model 3 was further adjusted for energy intake; model 4 was further adjusted for disease count, disability and swallowing problems

Table S9. Reaching protein thresholds according to physical activity level in community-dwelling older adults from the Netherlands (DNFCS), Finland (FINDIET) and Italy (INRAN-SCAI)

	Physical activity level			
	Low	Intermediate	High	p-value
DNFCS (n=1476 days)				
≥2 eating occasions >20g protein (%)				
Model 1	26.9 (13.0-40.9)	42.2 (35.3-49.1)	49.1 (45.9-52.3)	0.0035
Model 2	28.0 (13.7-42.2)	43.3 (35.7-50.9)	49.9 (45.7-54.2)	0.0043
Model 3	33.6 (20.5-46.8)	43.5 (36.5-50.5)	48.5 (44.5-52.4)	0.048
Model 4	34.8 (21.2-48.4)	43.3 (36.6-49.9)	48.5 (44.8-52.3)	0.061
Exclusion special days	34.2 (19.7-48.6)	43.2 (36.2-50.2)	48.3 (44.3-52.2)	0.079
Exclusion special diets	39.2 (24.0-54.4)	44.5 (37.1-52.0)	49.4 (45.4-53.4)	0.23
≥ 2 eating occasions >30g protein (%)				
Model 1	2.9 (-4.9-10.8)	8.3 (4.4-12.2)	10.3 (8.5-12.1)	0.16
Model 2	1.9 (-6.1-9.9)	7.7 (3.5-12.0)	9.8 (7.4-12.2)	0.12
Model 3	4.9 (-2.6-12.4)	7.8 (3.8-11.8)	9.0 (6.8-11.3)	0.50
Model 4	5.8 (-2.4-14.0)	7.8 (3.8-11.9)	9.0 (6.7-11.2)	0.67
Exclusion special days	3.7 (-4.7-12.1)	6.9 (2.8-10.9)	8.6 (6.3-10.9)	0.41
Exclusion special diets	5.7 (-3.7-15.1)	8.3 (3.7-12.9)	9.0 (6.6-11.5)	0.76
≥ 2 main meals >20g protein (%)				
Model 1	20.6 (6.7-34.6)	41.1 (34.2-48.0)*	46.2 (42.9-49.4)	0.0015
Model 2	20.5 (6.2-34.8)	41.5 (33.9-49.1)*	46.0 (41.7-50.3)	0.0018
Model 3	25.5 (12.1-38.9)	41.6 (34.5-48.7)*	45.7 (40.7-48.7)	0.018
Model 4	27.2 (13.3-41.1)	41.4 (34.6-48.3)	44.7 (40.9-48.6	0.039
Exclusion special days	26.6 (11.9-41.2)	41.3 (34.2-48.4)	44.6 (40.6-48.6)*	0.046
Exclusion special diets	28.9 (13.3-44.6)	42.7 (35.0-50.4)	45.4 (41.3-49.5)	0.11
≥ 2 main meals >30g protein				
Model 1	2.8 (-4.8-10.4)	6.8 (3.0-10.6)	9.7 (7.9-11.5)	0.11
Model 2	1.6 (-6.2-9.4)	6.0 (1.9-10.2)	9.0 (6.7-11.3)	0.089
Model 3	4.1 (-3.3-11.6)	6.1 (2.2-10.1)	8.3 (6.1-10.6)	0.34
Model 4	4.9 (-3.2-13.0)	6.1 (2.2-10.1)	8.3 (6.1-10.5)	0.45
Exclusion special days	2.7 (-5.6-11.0)	4.9 (0.9-9.0)	7.9 (5.6-10.2)	0.21
Exclusion special diets	4.5 (-4.8-13.8)	6.6 (2.1-11.2)	8.2 (5.7-10.6)	0.62
FINDIET (n=1734 days)				
≥2 eating occasions >20g protein (%)				
Model 1	34.0 (27.5-40.5)	37.2 (33.9-40.5)	35.3 (29.5-41.1)	0.65
Model 2	37.3 (29.4-45.1)	39.0 (33.4-44.6)	38.0 (30.3-45.7)	0.88
Model 3	38.6 (32.0-45.3)	40.1 (35.4-44.8)	36.4 (29.9-42.8)	0.42
Model 4	40.5 (34.2-46.8)	40.9 (36.5-45.4)	36.0 (29.9-42.1)	0.20
Exclusion special days	39.7 (33.2-46.2)	40.3 (35.7-44.9)	35.6 (29.2-41.9)	0.26

Table S9. Reaching protein thresholds according to physical activity level in community-dwelling older adults from the Netherlands (DNFCS), Finland (FINDIET) and Italy (INRAN-SCAI)

	**Physical activity level**			**p-value**
	**Low**	**Intermediate**	**High**	
≥ 2 eating occasions >30g protein (%)				
Model 1	12.3 (8.0-16.6)	10.6 (8.4-12.8)	12.2 (8.4-16.0)	0.68
Model 2	13.6 (8.4-18.9)	11.9 (8.2-15.7)	14.6 (9.5-19.7)	0.47
Model 3	14.5 (10.0-18.9)	12.6 (9.4-15.8)	13.6 (9.2-17.8)	0.66
Model 4	14.4 (9.9-18.9)	12.6 (9.4-15.8)	13.6 (9.2-18.0)	0.66
Exclusion special days	15.3 (10.7-19.9)	12.8 (9.5-16.0)	13.7 (9.2-18.2)	0.52
≥ 2 main meals >20g protein (%)				
Model 1	24.8 (18.7-31.0)	27.5 (24.4-30.7)	26.6 (21.1-32.0)	0.74
Model 2	27.2 (19.8-34.6)	28.3 (23.1-33.6)	27.6 (20.4-34.8)	0.94
Model 3	28.2 (21.5-34.8)	29.1 (24.4-33.9)	26.5 (20.0-33.0)	0.66
Model 4	29.4 (23.0-35.9)	29.7 (25.1-34.3)	26.2 (19.9-32.5)	0.47
Exclusion special days	28.3 (21.7-35.0)	29.2 (24.5-33.9)	26.4 (19.8-32.9)	0.62
≥ 2 main meals >30g protein (%)				
Model 1	8.4 (4.7-12.2)	8.2 (6.2-10.1)	7.8 (4.5-11.2)	0.97
Model 2	9.2 (4.60-13.8)	8.4 (5.2-11.7)	8.5 (4.1-13.0)	0.95
Model 3	9.7 (5.6-13.9)	8.9 (5.9-11.9)	7.9 (3.8-11.9)	0.74
Model 4	9.7 (5.6-13.9)	8.9 (5.9-11.8)	7.9 (3.8-11.9)	0.75
Exclusion special days	10.2 (5.9-14.5)	8.9 (5.9-11.9)	7.8 (3.6-12.0)	0.64
INRAN-SCAI (n=1530 days)				
≥2 eating occasions >20g protein (%)				
Model 1	63.8 (55.8-71.8)	63.5 (59.0-68.0)	70.0 (65.5-74.4)	0.11
Model 2	58.2 (47.3-69.1)	56.7 (47.7-65.7)	62.0 (52.8-71.2)	0.24
Model 3	58.0 (49.0-67.1)	56.3 (48.9-63.8)	58.0 (50.4-65.6)	0.80
Model 4	60.8 (52.4-69.2)	60.2 (53.3-67.2)	62.2 (55.1-69.3)	0.72
Exclusion special days	62.7 (54.0-71.4)	61.1 (54.1-68.1)	62.6 (55.4-69.7)	0.83
Exclusion special diets	60.5 (51.9-69.0)	61.0 (54.1-68.0)	62.4 (55.3-69.4)	0.82
≥ 2 eating occasions >30g protein (%)				
Model 1	31.1 (23.5-38.7)	33.6 (29.3-37.8)	35.7 (31.4-40.0)	0.56
Model 2	28.5 (18.1-38.9)	30.1 (21.5-38.7)	31.8 (21.5-38.7)	0.73
Model 3	28.3 (20.1-36.6)	29.7 (22.9-36.5)	27.4 (20.4-34.3)	0.62
Model 4	29.0 (20.7-37.2)	30.6 (23.8-37.4)	28.3 (21.4-35.3)	0.63
Exclusion special days	30.6 (21.8-39.5)	31.0 (23.9-38.2)	29.1 (21.9-36.3)	0.75
Exclusion special diets	28.4 (19.8-37.0)	31.7 (24.8-38.6)	29.4 (22.3-36.4)	0.53
≥ 2 main meals >20g protein (%)				
Model 1	62.7 (54.6-70.7)	63.2 (58.7-67.7)	69.7 (65.2-74.2)	0.096

Table S9. Reaching protein thresholds according to physical activity level in community-dwelling older adults from the Netherlands (DNFCS), Finland (FINDIET) and Italy (INRAN-SCAI)

	**Physical activity level**			**p-value**
	**Low**	**Intermediate**	**High**	
Model 2	57.3 (46.3-68.3)	56.6 (47.5-65.7)	61.9 (52.6-71.2)	0.23
Model 3	57.1 (48.0-66.3)	56.2 (48.6-63.8)	57.9 (50.2-65.6)	0.81
Model 4	59.9 (51.3-68.4)	60.0 (52.9-67.1)	62.1 (54.9-69.3)	0.67
Exclusion special days	61.6 (52.6-70.5)	61.1 (53.9-68.2)	62.5 (55.2-69.8)	0.87
Exclusion special diets	59.3 (50.6-68.1)	60.8 (53.8-67.9)	62.2 (55.1-69.4)	0.72
≥ 2 main meals >30g protein				
Model 1	31.1 (23.5-38.8)	33.4 (29.2-37.7)	35.6 (31.3-39.8)	0.57
Model 2	28.5 (18.0-38.9)	29.9 (21.3-38.6)	31.6 (22.8-40.4)	0.74
Model 3	28.3 (20.1-36.6)	29.5 (22.7-36.4)	27.2 (20.3-34.2)	0.63
Model 4	29.0 (20.7-37.2)	30.4 (23.6-37.2)	28.2 (21.2-35.2)	0.65
Exclusion special days	30.7 (21.8-39.6)	31.1 (23.9-38.2)	29.0 (21.7-36.3)	0.72
Exclusion special diets	28.4 (19.8-37.1)	31.5 (24.5-38.5)	29.2 (22.1-36.3)	0.56

Number of meals reaching protein thresholds presented as % (95%-CI).
Model 1: adjusted for age and sex. Model 2: adjusted for age, sex, educational level, marital status, and smoking. Model 3: Model 2 additionally adjusted for energy intake. Model 4: model 3 additionally adjusted for protein intake status (<0.8 g/kg aBW/day).

Table S10. Number of main meals providing ≥20 or 30 grams of protein according to sedentary behaviour in community-dwelling older adults from Finland (FINDIET) and Italy (INRAN-SCAI)

	Sedentary behaviour			
	**<4 hrs/day**	**4-6 hrs/day**	**>6 hrs/day**	**p-value**
FINDIET (n=1596)				
≥2 eating occasions >20 g (%)				
Model 1	38.9 (34.0-43.7)	34.4 (30.3-38.5)	36.8 (31.0-42.7)	0.39
Model 2	38.3 (30.9-45.7)	32.9 (26.0-39.8)	34.6 (26.6-42.6)	0.26
Model 3	39.0 (32.8-45.2)	33.0 (27.2-38.8)	35.1 (28.4-41.8)	0.096
Excluding special days	39.1 (33.0-45.1)	32.8 (27.1-38.5)	35.0 (28.5-41.6)	0.068
Excluding special diets	N/A	N/A	N/A	
≥ 2 main meals >20 g (%)				
Model 1	28.5 (23.9-33.0)	25.8 (21.9-29.6)	28.4 (22.8-33.9)	0.60
Model 2	26.6 (19.6-33.6)	23.4 (16.9-30.0)	25.5 (18.0-33.1)	0.58
Model 3	27.1 (20.9-33.4)	23.5 (17.7-29.4)	25.9 (19.1-32.6)	0.42
Excluding special days	26.8 (20.6-33.1)	23.3 (17.4-29.2)	25.4 (18.6-32.2)	0.44
Excluding special diets	N/A	N/A	N/A	
≥ eating occasions >30 g (%)				
Model 1	9.9 (6.7-13.1)	11.9 (9.1-14.6)	11.2 (7.3-15.1)	0.65
Model 2	10.3 (5.3-15.2)	12.9 (8.3-17.5)	12.2 (6.9-17.6)	0.48
Model 3	10.7 (6.5-15.0)	13.0 (9.1-17.0)	12.5 (7.9-17.1)	0.47
Excluding special days	10.7 (6.4-15.0)	13.3 (9.2-17.3)	13.3 (8.6-18.0)	0.36
Excluding special diets	N/A	N/A	N/A	
≥ 2 main meals >30 g (%)				
Model 1	8.2 (5.3-11.0)	7.9 (5.5-10.3)	9.1 (5.7-12.6)	0.85
Model 2	7.4 (3.0-11.7)	7.8 (3.7-11.9)	8.8 (4.1-13.5)	0.82
Model 3	7.7 (3.7-11.6)	7.9 (4.2-11.6)	9.0 (4.7-13.3)	0.80
Excluding special days	7.5 (3.5-11.6)	7.8 (4.0-11.6)	9.7 (5.3-14.0)	0.58
Excluding special diets	N/A	N/A	N/A	
INRAN-SCAI (n=1479)				
≥2 eating occasions >20 g (%)				
Model 1	58.2 (50.7-65.6)	69.5 (64.2-74.8)	67.9 (63.8-72.1)	0.039
Model 2	51.9 (43.2-60.6)	63.9 (56.9-71.0)	62.2 (56.0-68.4)	0.025
Model 3	52.3 (45.2-59.4)	61.5 (55.7-67.3)	61.7 (56.7-66.8)	0.023
Excluding special days	59.0 (0.52-0.66)	62.9 (57.2-68.5)	65.4 (60.5-70.4)	0.19
Excluding special diets	54.2 (47.5-60.9)	62.7 (57.2-68.2)	65.3 (60.4-70.2)	0.0052
≥ 2 main meals >20 g (%)				
Model 1	58.1 (50.6-65.6)	69.1 (63.7-74.5)	67.4 (63.2-71.6)	0.051
Model 2	52.0 (43.2-60.7)	63.5 (56.4-70.6)	61.7 (55.4-70.0)	0.038
Model 3	52.4 (45.2-59.6)	61.1 (55.2-66.9)	61.2 (56.1-66.4)	0.039
Excluding special days	59.1 (51.8-66.4)	62.3 (56.6-68.1)	65.1 (60.0-70.2)	0.24
Excluding special diets	54.3 (47.4-61.1)	62.2 (56.6-67.8)	64.6 (59.7-69.6)	0.012
≥ 2 eating occasions >30 g (%)				
Model 1	27.2 (20.2-34.3)	32.9 (27.8-37.9)	37.4 (33.5-41.4)	0.042
Model 2	23.5 (15.2-31.7)	29.8 (23.1-36.4)	33.6 (27.8-39.5)	0.046

Table S10. Number of main meals providing ≥20 or 30 grams of protein according to sedentary behaviour in community-dwelling older adults from Finland (FINDIET) and Italy (INRAN-SCAI)

	Sedentary behaviour			
	**<4 hrs/day**	**4-6 hrs/day**	**>6 hrs/day**	**p-value**
Model 3	23.9 (17.5-30.4)	27.2 (21.9-32.4)	33.1 (28.5-37.8)	0.0063
Excluding special days	26.1 (18.9-33.3)	28.7 (23.0-34.3)	34.3 (29.3-39.3)	0.029
Excluding special diets	24.1 (17.4-30.7)	27.9 (22.4-33.4)	34.7 (29.9-39.6)	0.0019
≥ 2 main meals >30 g (%)				
Model 1	27.2 (20.2-34.3)	32.9 (27.8-37.9)	37.2 (33.2-41.1)	0.050
Model 2	23.4 (15.2-31.7)	29.7 (23.1-36.4)	33.3 (27.4-39.2)	0.056
Model 3	23.9 (17.4-30.4)	27.2 (21.9-32.4)	32.8 (28.2-37.5)	0.0094
Excluding special days	26.2 (18.9-33.4)	28.7 (23.0-34.4)	34.3 (29.2-39.3)	0.033
Excluding special diets	24.0 (17.3-30.7)	27.9 (22.3-33.4)	34.4 (29.5-29.3)	0.0031

Presented as % (95%-CI). Model 1: adjusted for age and sex. Model 2: adjusted for age, sex, educational level, marital status, household income (except for INRAN-SCAI) and smoking. Model 3: Model 2 additionally adjusted for energy intake. N/A Not available

Physical activity and pattern of protein intake over the day

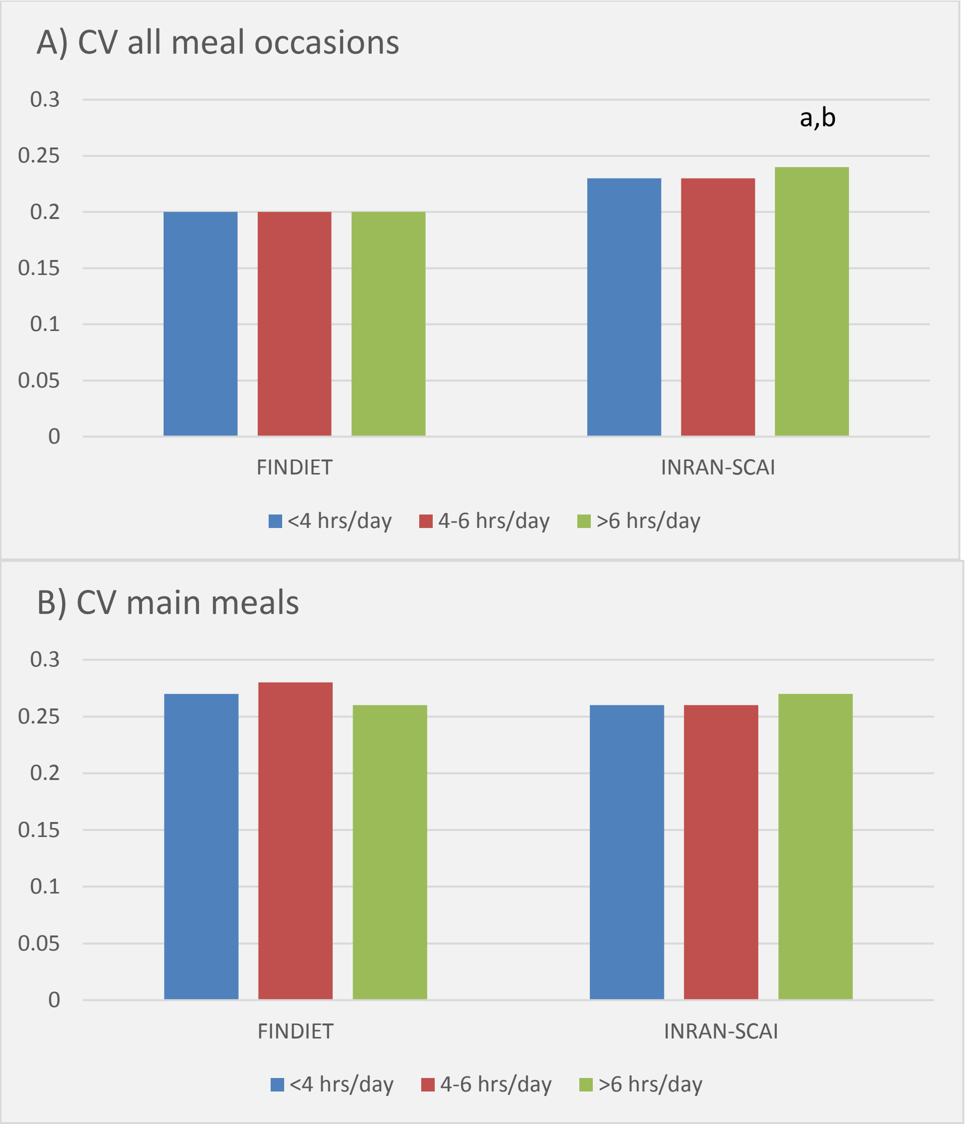

Figure S4. Coefficient of variation of protein intake over A) all eating occasions or B) main meals only according to sedentary behaviour in Finland (FINDIET) and Italy (INRAN-SCAI). *A lower CV indicates a more evenly distributed protein intake. Adjusted for age, sex, educational level, marital status, and smoking.* [a] *Differs from those with < 4 hrs sedentary behaviour per day p<0.05.*[b] *Differs from those with 4-6 hrs sedentary behaviour per day p<0.05.*

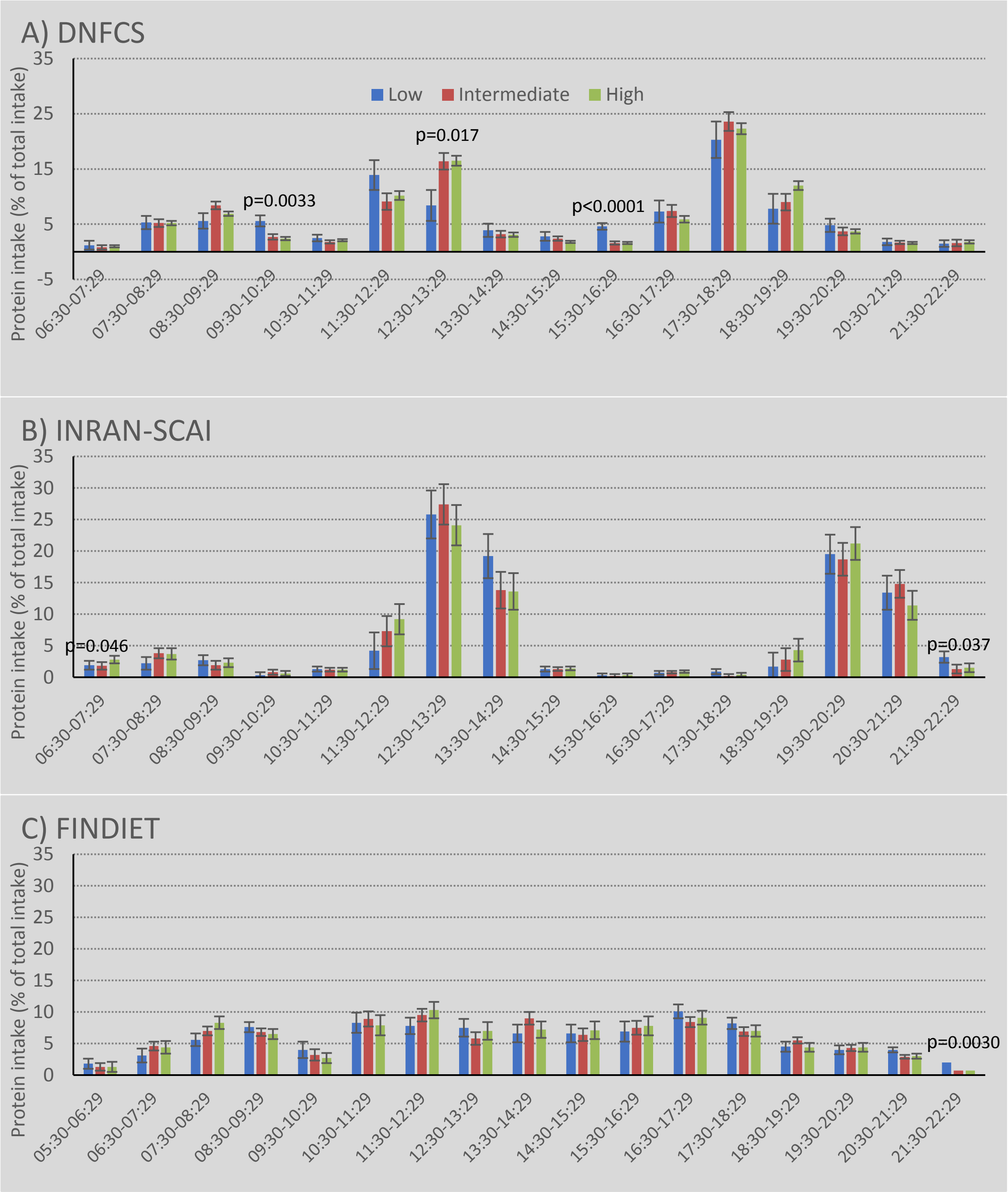

Figure S5. Proportion of protein intake (% of total intake) across time of the day and stratified by physical activity level among community-dwelling older adults in A) the Netherlands (DNFCS), B) Italy (INRAN-SCAI), and C) Finland (FINDIET). *Adjusted for age, sex, educational level, marital status, household income, and smoking.*

References

1. Mendonça, N., et al., *Prevalence and determinants of low protein intake in very old adults: insights from the Newcastle 85+ Study.* European journal of nutrition, 2018. **57**(8): p. 2713-2722.

2. Granic, A., et al., *Dietary patterns and socioeconomic status in the very old: the Newcastle 85+ Study.* PloS one, 2015. **10**(10).

3. Noble, M., M.E. Wilkinson, and M.H. Barnes, *The English indices of deprivation 2007.* 2008.

4. Salmond, C., P. Crampton, and J. Atkinson, *NZDep2006 Index of Deprivation*, U.o.O. Department of Public Health, Editor. 2007, Ministry of Health: Wellington.
